# A bibliometric analysis and mapping of global research trends in antihypertensive medication adherence (1975–2024)

**DOI:** 10.1186/s43044-025-00681-9

**Published:** 2025-09-03

**Authors:** Mokanpally Sandeep, M. Surya Durga Prasad, Sree Sudha Tanguturi Yella, Dandge Shailendra, B. R. Shamanna

**Affiliations:** 1 School of Medical Sciences, University of Hyderabad, Hyderabad, India; 2 All India Institute of Medical Sciences, Deoghar, Jharkhand India; 3 Mediciti Institute of Medical Sciences, Hyderabad, India

**Keywords:** Hypertension, Antihypertensives, Medication adherence, Patient compliance, Bibliometric analysis

## Abstract

**Background:**

Hypertension, a significant risk factor for cardiovascular diseases, has increased dramatically over the decades. It can be mitigated with proper medication utilization and lifestyle modifications. Adherence to antihypertensive medication plays a significant role in reducing severe complications and improving the overall quality of life of people living with hypertension. This study was conducted to assess the global research output and trends in antihypertensive medication adherence.

**Methods:**

The study utilized the Scopus database for bibliometric analysis. The keywords related to antihypertensive medication adherence were searched from the database’s inception to 21st December 2024. The retrieved data were analysed using VOSviewer, Biblioshiny and QGIS 3.30.1. The bibliometric maps and indicators were presented based on publication and funding source, most contributed countries and authors, keyword co-occurrence, and other relevant and important indicators.

**Results:**

In total, 3497 documents specifically related to antihypertensive medication adherence were utilized and they were identified from 1975 to 2024. The publication’s annual growth rate was identified as 8.65%. The USA (1144, 33%), the UK (269, 8%), and Germany (246, 7%) are the leading contributors to antihypertensives adherence research. Schmieder R.E was found to be the top published author in this domain. The Journal of Hypertension was identified as the most published source, while National Institute of Health (NIH) revealed as the top funding body supporting antihypertensives adherence research.

**Conclusions:**

Research on antihypertensive medication adherence was largely contributed by developed countries, especially dominated by the USA in most of the indicators. As the contribution from developing countries is limited, LMICs must be prioritized with collaborative research and capacity building to strengthen this adherence research. These efforts gradually help countries to generate context-specific evidence to improve adherence to antihypertensive medications thereby reducing the global burden of hypertension.

**Supplementary Information:**

The online version contains supplementary material available at 10.1186/s43044-025-00681-9.

## Background

Hypertension stands as a leading contributor to mortality resulting from non-communicable chronic diseases (NCDs) on a global scale [1–3]. The prevalence of hypertension in the worldwide population reaches 22%, impacting a substantial number of individuals. Annually, around 9.4 million deaths are attributed to complications arising from this condition [4]. In the context of these environments, promoting health faces a significant hurdle in ensuring both medical and non-medical adherence to hypertension treatments. The prevalence of complications linked to hypertension is consistently increasing in middle- and low-income countries, driven in part by an ageing population and the imperative to embrace healthier lifestyles [5, 6]. A noteworthy public health issue arises from the substantial non-compliance with treatment regimens, with half of the patients neglecting to adhere to prescribed medical intervention [7–10].

Despite significant progress in therapeutic interventions and public health initiatives, there is still room for improvement in the awareness, treatment, and management of hypertension, with notable variations observed among different countries [11]. In essence, on one hand the developed countries are addressing the needs of the population suffering with hypertension with all essential medical resources, on the other hand developing countries though tackling with limited resources, trying to provide their best care to the people living with hypertension. In either situation, there is still a substantial need for health education and awareness, especially treatment adherence to these modern NCD epidemics to reduce its associated morbidity and mortality. The substantial global impact of hypertension has incited extensive research efforts across various medical and scientific disciplines over the past few decades. This surge in research has given rise to specialized journals like Hypertension [12] and the Journal of Hypertension [13], both established in 1979 and 1983, respectively. Research on adherence to treatment has revealed opportunities for developing new strategies and behavioural interventions. These efforts focus on ensuring the proper monitoring of prescribed therapies, which in turn can significantly improve patients’ quality of life [14, 15]. The interventions include health promotion initiatives that encourage behaviour changes on both individual and collective levels, tailored to the social contexts of the individuals involved. The overarching goal is to enhance adherence to non-communicable disease (NCD) therapy [16, 17].

## Objective

Given the limitation of lack of bibliometric analysis in existing literature, the current study was undertaken with an objective to evaluate the worldwide research output and prevailing patterns related to adherence to antihypertensive medication.

## Methodology

### Choice of database

For this bibliometric analysis, the research team selected a single database to source the relevant literature for thorough quantitative evaluation. We chose Scopus due to its distinct benefits compared to other databases such as Medline and Web of Science. Scopus not only encompasses the entirety of Medline’s content but also extends beyond the reach of Web of Science in terms of scope. With more than 23,000 indexed journals covering medical, social, and scientific disciplines, Scopus offers a comprehensive and diverse dataset ideal for our analysis [18, 19]. Additionally, Scopus offers a range of user-friendly features specifically designed to facilitate bibliometric analysis, enhancing efficiency and accessibility for researchers.

### Search strategy

The search strategy utilized a comprehensive approach to identify relevant literature on antihypertensive medication adherence. It employed a range of keywords and phrases related to adherence, including variations such as ‘medication adherence’, ‘compliance’, and specific terms like ‘hypertensive medication adherence’ and ‘antihypertensives adherence.’ We used the Boolean operator “OR” for combining similar words in a single search strategy arm and the Boolean operator “AND” is used to combine separate search strategies to get final results. By focusing on titles and abstracts, the search aimed to capture relevant studies spanning from the inception of the database to the present (21st December, 2024). Studies published in all languages and published as article, review, book chapter, conference paper and short survey were considered eligible for the study inclusion. Papers published as letter, editorial, note, retracted were considered for exclusion. For articles published in non-English language, bibliometric information translated in English language was available in the database such as title, abstract, keywords, and citation metrics. This approach ensures a broad coverage of literature in the field of study, facilitating a comprehensive understanding of medication adherence across various contexts and populations. The search strategy was available in MS Word format as Supplementary file (Table S1).

### Screening and bibliometric indicators and mapping

The retrieved articles were undergone for a review to identify the documents which are not in line with inclusion criteria as mentioned in table S1. After eliminating the letters, editorials, notes and retracted, the resulting documents were imported for the bibliometric analysis. In this study, we utilized a comprehensive approach to analyse the retrieved data. We employed VOSviewer, R programming software 4.3.2 and QGIS 3.30.1 to conduct our analysis. Through these tools, we were able to generate bibliometric maps and indicators, shedding light on various aspects of the research landscape. Our analysis encompassed several key bibliometric indicators and mapping techniques. These included examining the publication annual growth rate, average citations per document and per year, affiliations of authors, authors’ co-authorship patterns, countries and authors making the most significant contributions, keyword co-occurrence patterns, the most frequently published sources, and the primary funders of research in the field. By employing these methods and metrics, we gained valuable insights into the trends, patterns, and stakeholders shaping the research landscape, providing a comprehensive understanding of the field’s dynamics and contributions.

## Results

**Fig. 1:**
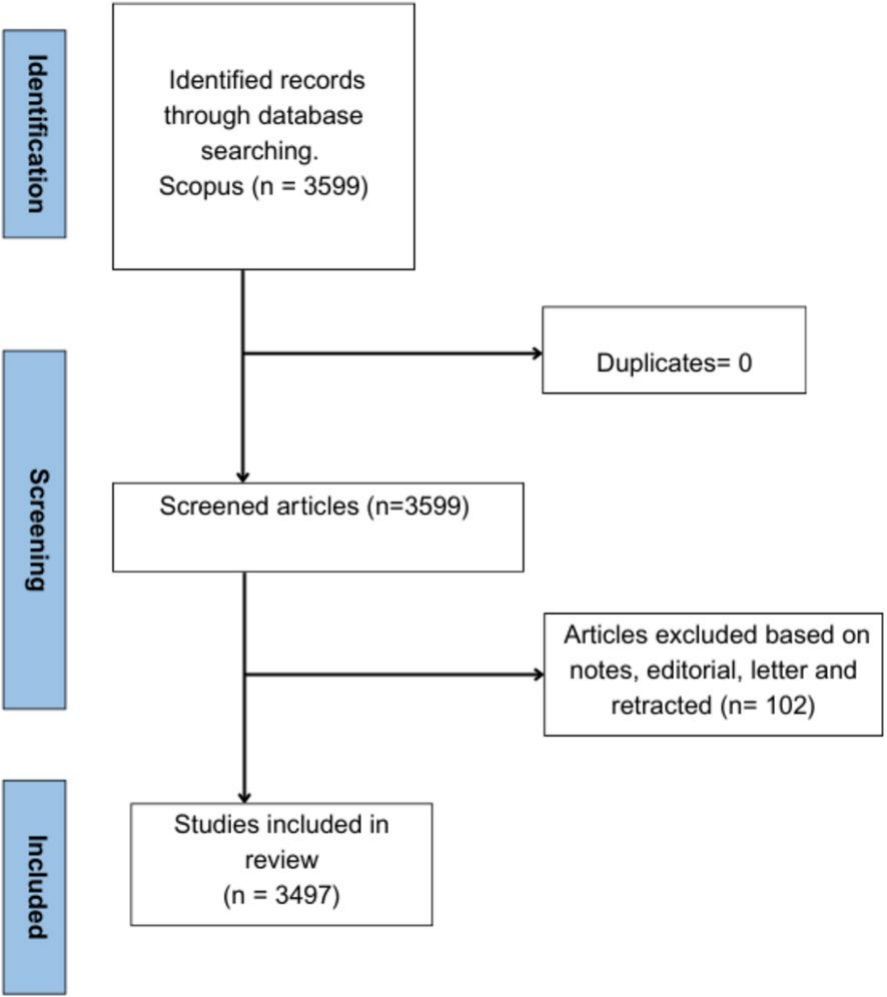
Flow chart of articles identification and inclusion

A total of 3599 documents were retrieved from Scopus database and are identified from 1975 to 2024. The majority of the retrieved documents were journal articles 2625 (290 trails), followed by Review 755, Conference paper 72, Note 44, Editorial 41, Book chapter 16, Short survey 29, Letter 15, and Retracted 2. The study did not consider the letter, notes, editorials and retracted for the robustness of the study findings. Hence, a total of 3497 documents were utilized in the final analysis for this study (Fig. 1). The retrieved data were reported from 1050 sources. Majority of the documents reported in English (89%), Spanish (2.3%) and Germany (2.2%). Annual growth rate of the documents was 8.65%. Average citation per document was identified as 40.03. The focus on emphasizing the critical importance of medication adherence for hypertension management was first well described by Haynes et al., in the year 1976 which was featured in LANCET [20].

### Most frequently used author keywords

**Fig. 2:**
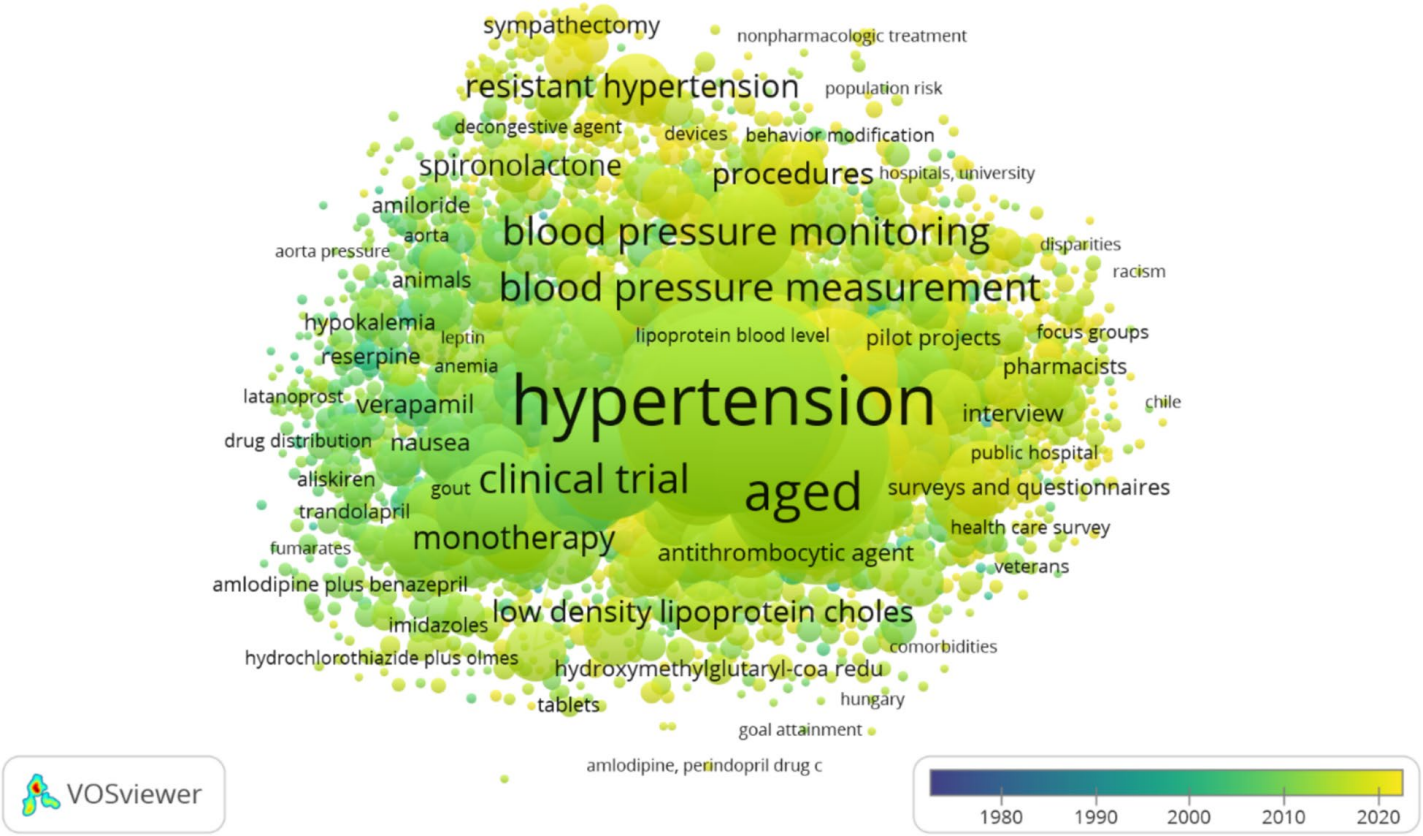
Co-occurrence of keywords in antihypertensive medication adherence research

The study employed a network map to visualize the most commonly used author keywords (Fig. 2). A total of 122,403 key words were identified but to identify the most prominent keywords in the discipline a threshold of minimum 5 times occurrence was considered. 2677 keywords met the criteria for the analysis. In this map, the size of each node indicates the frequency with which each keyword appears. Apart from adherence and compliance, most frequently reported keywords are blood pressure regulation, treatment outcome, clinical study and dipeptidyl carboxypeptidase inhibitor. This approach offers a clear and intuitive representation of the prominent themes or concepts addressed within the study’s literature.

### International and national collaboration

**Fig. 3:**
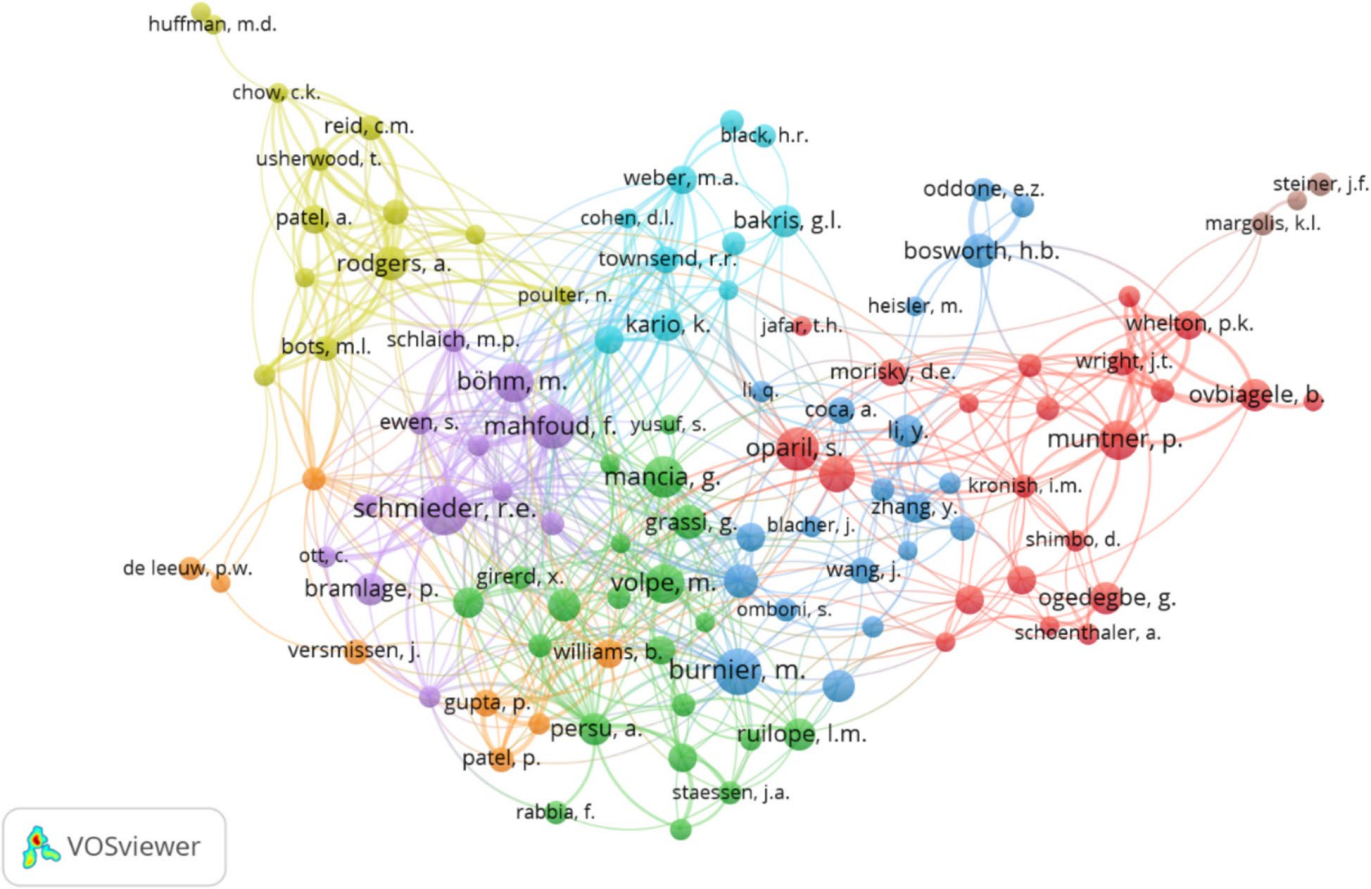
Authors co-authorship in antihypertensive medication adherence research

The co-authorship network visualization map revealed the collaborative patterns among active authors, showcasing the strength of research collaborations between them. Utilizing VOSviewer, the study identified 13,789 authors but by keeping a criterion of minimum 8 documents by an author 109 authors were considered for the analysis. This criterion was undertaken to reduce the ambiguity and to provide better a representation of the most publishing authors collaborations. The resulting figure displayed a density visualization map of co-authorship based on authors (Fig. 3). 109 authors, each with a minimum of 8 documents, were categorized into 8 clusters, amounting to a total of 604 links with a cumulative link strength of 1403. These clusters were denoted by different colours, including red, green, blue, yellow, purple, sea blue, orange, and brown consisting of 22, 21, 20, 13, 12, 10, 8, and 3 authors, respectively. Observations indicated that most of the top-collaborating authors hailed from the USA and Germany, emphasizing the significance of their contributions to the collaborative research landscape.

### Growth of publications and citation analysis

The exploration of antihypertensive medication adherence in scientific literature began in 1975, with publication numbers remaining relatively low until 2007. However, a significant uptick in publications became apparent thereafter. Prior to 2006, annual scientific production consistently fell below 100 documents. From 2007 onward, there was a consistent increase, with more than 100 publications per year. The peak year for publications was 2017, with a record high of 190 documents. This trend in publication growth is visually depicted in Figure S1.

**Fig. 4:**
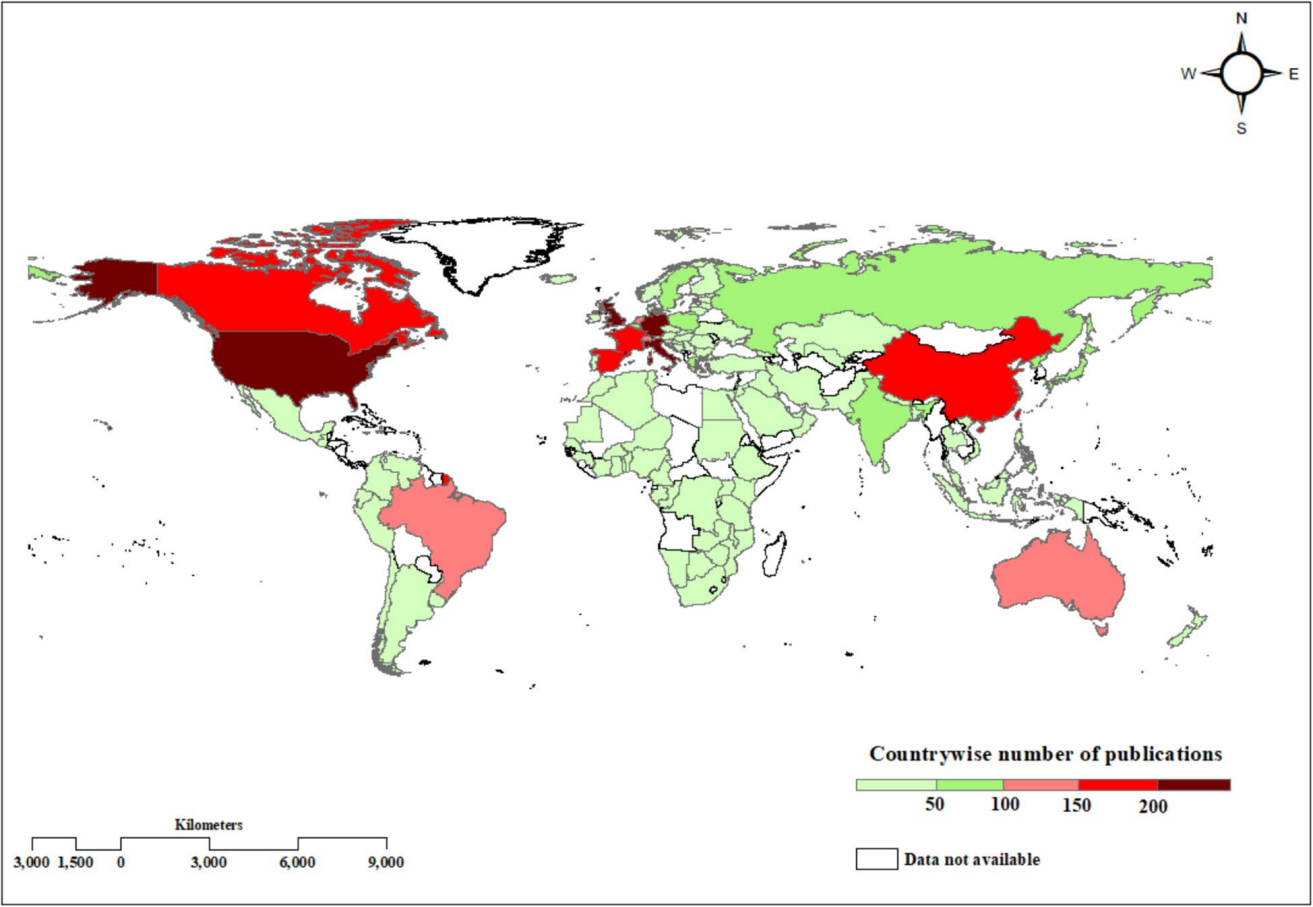
Distribution of global publications depicted by choropleth map by country between 1975 and 2024 as identified in Scopus until 21st December 2024. The map was created using QGIS 3.30.1

The countries with the highest contributions in terms of document count were the USA, the UK, Germany, and Italy, with 1144 (33%), 269 (8%), 246 (7%), and 233 (6%) documents, respectively (Table S2). Further, the choropleth map utilized the data from 119 countries depicting the largest share of documents hailed from developed economies. The QGIS map allowed us to identify the number of research publications represented by each country by varying colour shades. Scholarly article distribution across different regions is better represented in this map, allowing a greater understanding of both visual and spatial insights (Fig. 4).

Citation frequency in scientific publications serves as a barometer for gauging the impact of an article on its subject matter, as well as on its authors and the journals in which it’s published. Among a total of 3497 articles analysed, the average citations per document stood at 40.03. Notably, the highest average citations in a single year were recorded in 2018, reaching 17.51 (Figure. S2). These figures underscore the varying degrees of impact and recognition within the academic community.

### Active journals or sources published most in antihypertensive medication adherence

Table 1 lists the leading journals for publishing research on adherence to antihypertensive medication, drawing from a total of 1050 sources. Notably, among the active journals, the ‘Journal of Hypertension’ secured 1st position within the top 10. However, ‘Hypertension’ journal reported to have the highest average citations per document (193; h-index 7.2), revealing its impact on antihypertensive medication adherence research.

**Table 1:** Top 10 most contributed sources in antihypertensive medication adherence research

S.No	Source name	Number of documents	Total citations	Average citations per document	h-index	Current impact factor of the journal
1	Journal Of hypertension	99	9924	100	42	3.3
2	Journal Of clinical hypertension	95	1966	21	26	2.7
3	American journal of hypertension	78	4319	55	35	3.2
4	Journal of human hypertension	74	3287	44	29	2.7
5	Hypertension	68	13,142	193	40	7.2
6	Current hypertension reports	56	1397	25	22	3.9
7	High blood pressure and cardiovascular prevention	41	442	11	13	3.1
8	PLOS one	38	998	26	16	2.9
9	Patient preference and adherence	35	710	20	16	2.0
10	Trials	31	425	14	12	2.0

Table 2 presents the top 10 funding sources for research on antihypertensive medication adherence. Funding in this area was contributed by 159 sources across the world. Top 5 leading funding contributors are hailed from the USA. Notably, the National institute of health emerged as the primary funding source for this area, providing funding on 200 occasions. Research supported from this institute is also shown to achieve the highest average citations (45) and h-index (50), exemplifying its significant contribution to advancing research in this field.

**Table 2:** Top 10 funding sources for adherence to antihypertensive medication research

S.No	Source title	Number of documents	Total citations	Average citations per document	h-index
1	National institutes of health	200	8969	45	50
2	National heart, lung, and blood institute	153	7939	52	45
3	U.S department of health and human services	140	7122	51	43
4	National institute of diabetes and digestive and kidney diseases	48	2034	42	21
5	National center for advancing translational sciences	38	1115	30	19
6	Pfizer	32	1764	55	21
7	National health and medical research council	27	1015	37	16
8	Medtronic	30	1967	66	17
9	National natural science foundation of china	28	395	14	11
10	National institute of aging	28	2712	97	19

Table 3 displays the top 10 active authors, with notable contributions from Schmieder, R.E. topping the list. It is important to note that all the five leading contributing authors are hailed from developed countries such as Germany, the USA, Switzerland and Italy. These authors have made a significant contribution to the field, reflecting their extensive involvement in antihypertensive medication adherence research endeavours. VA medical centre emerges as the leading affiliation of authors contributed most in this field, with an impressive output of 67 documents with an h-index-34 of the published documents. The University of Alabama at Birmingham tops in highest total number of citations (20137). This institution’s prolific output underscores its pivotal role in advancing research within its domain (Table S.3).

**Table 3:** Top 10 most contributed authors to antihypertensive medication adherence research

S.No	Author name	Number of documents	Total citations	Average citations per document	h_index
1	Schmieder, R.E	39	4912	126	22
2	Mahfoud, F	35	5585	160	21
3	Oparil, S	33	19,589	593	19
4	Burnier, M	31	5261	170	20
5	Mancia G	30	4020	134	20
6	Böhm, M	27	2444	90	17
7	Muntner, P	25	13,380	535	20
8	Parati G	22	1307	60	15
9	Kario, K	22	2969	135	16
10	Azizi, M	19	4239	223	15

## Discussion

This bibliometric analysis examines global research trends in antihypertensive medication adherence using the Scopus database, chosen for its comprehensive coverage extending beyond Medline and Web of Science. While acknowledging the influence of database choice and search strategy on results, our findings reveal a significant rise in publications from 2007, averaging approximately 100 new publications annually. A very few studies using bibliometric data were conducted focusing on pharmacy-related issues [21–23]; this study is a new addition to them with special focus on antihypertensives context. The increased interest in hypertension research resulting in the research publications can be attributed to the growing burden of hypertension both in terms of morbidity and mortality across the world [24, 25]. Of the many ways, medication adherence stands as one of the foundational elements in mitigating hypertension risk. Nonadherence to the antihypertensive medication resulting in severe cardiovascular outcomes was well documented [26, 27]. This led to the critical understanding of the psychosocial and behavioural factors influence on the medication adherence.

In the recent decades, substantial contribution of research in this field has been reinforced with a number of review papers established. Our analysis revealed nearly 755 review articles interrogating the importance of various aspects related to the antihypertensive medication adherence. Exploring evidence with substantial literature is quintessential in identifying and addressing the major gaps in any field. The current data revealed a substantial contribution with cross sectional and observational studies, but only 290 trials were conducted to identify the different lacuna in antihypertensive medication adherence across the world. This exposes the further need of conducting trials on this matter.

Regarding the geographical focus of research on antihypertensive interventions, the majority of scientific studies have been conducted in the USA, with significant contributions from the UK as well. The USA leads in both publication volume and collaborative research. The choropleth map exemplifies that the majority of the research publications are hailed from developed countries. Important to note that in the top 10 leading countries there is only China reporting from Asia standing in 9th position and no representation from either Africa or Middle East. In the case of Asia, there is a dire need of research on antihypertensive medication adherence as this region is substantially impacted by the NCDs, especially hypertension in the recent decades [28]. Researchers from these developing countries might explore in this domain as given the opportunities but challenges related to resources and language always prevailed in these regions hindering their research development in this field.

Authors and institutions in the USA, particularly those affiliated with VA Medical Centres, have made prominent contributions to this field. This focus is not coincidental, given the substantial burden of hypertension in the USA, where approximately one in 3 individuals, or 75.2 million Americans, are affected by this condition [29, 30]. This increase in documents can also be attributed to digital interventions as in recent years developed countries have largely tried to implement these interventions which includes SMS text messages, web platforms, apps, and WhatsApp which have gained increasing attention in adherence studies [31–34]. These technological tools not only provide health information but also aim to enhance patients’ overall quality of life [35]. This underscores the importance of ongoing research efforts, particularly within the USA.

The current study reveals a notable increase in publications over the last decade, particularly highlighting the Journal of Hypertension's significant focus on antihypertensive medication adherence, reflecting a growing awareness and interest in this critical area of hypertension management. Literature highlights the different evolving themes including telephone-based interventions which have proven to be effective, especially for patients with multiple comorbidities, by monitoring through calls [36–38]. Pharmacists in community pharmacies and clinics often use this approach, providing essential guidance on health behaviours, which significantly improves adherence to both medical and non-medical aspects of antihypertensive therapy [39]. In addition, emerging research also started focusing on the role of psychosocial and behavioural determinants influence on the antihypertensive medication adherence and it was found to be substantial [40, 41]. Treating these factors will eventually benefit the patients to become adherent to their medication regimen.

Hypertension research spans numerous specialties and journals. Observed publication trends may reflect developments within specific research areas. Given the need for an increase in collaborative research, analysing international investigator networks is crucial. Finally, with the significant proportion of the global hypertension burden concentrated in low- and middle-income countries, future research should prioritize these regions. Continuous global monitoring of hypertension research is essential for progress in this field.

### Strengths

This is the first ever bibliometric analysis to quantify the research trends in antihypertensive medication adherence at a global level. In addition, the study followed a screening process to eliminate the erroneous entries in final analysis. Furthermore, to increase the robustness of the study findings, 3 different data analysis programmes were used for the accurate bibliometric figures and tables.

### Limitations

The methodology employed in this study aimed to enhance result validity through the utilization of the extensive Scopus database. However, it has some limitations as it is noteworthy that a predominant number of indexed journals in Scopus are affiliated with the USA, the UK, and other English-centric research communities which may create an English language and global representation bias. In addition, there might be articles not indexed in Scopus but indexed in other databases such as PubMed and Web of Science, which remained uncovered in this analysis. Furthermore, Scopus offers English-language titles and abstracts for the majority of publications, though this analysis is solely dependent on bibliometric information which is available in English translation, the full-text material of studies that were not in English was not evaluated, which may restrict interpretation of evidence from local-language sources. Consequently, the findings related to authors’ and institutions may exhibit a certain skewness towards countries with a prevalence of Scopus-indexed journals.

This analysis also considered books and chapters which may have different quality, peer review standards and citation patterns gives rise to variability in metrics and can impact overall bibliometrics. Future studies may consider these limitations to do more comprehensive work. Despite these considerations, it is crucial to recognize the positive impact of global advancements in science and technology, along with the increasing number of scholars and academic institutions worldwide. These factors collectively contribute to the overall growth observed in publications on antihypertensive medication adherence.

## Conclusion

This bibliometric analysis brings to light notable gaps in research in antihypertensive medication adherence. In recent years, there has been a noticeable surge in interest and focus on research related to this field. The escalating number of publications annually underscores the growing significance of this research domain. The challenges anticipated in hypertension management and research in the developing regions may be expected to mirror those experienced in high-income countries over the last 50 years. Since global tracking of hypertension publications could prove beneficial for all stakeholders, all countries, especially developing countries, should be encouraged to collaborate and participate to improve the research trends in this field.

## Supplementary Information

Supplementary file 1.

## Data Availability

No datasets were generated or analysed during the current study.

## Abbreviations

GBD: Global burden of disease study
NCD: Non-communicable diseases
WHO: World health organization
SMS: Short message service
