# Supplementary material for: A bibliometric analysis and mapping of global research trends in antihypertensive medication adherence (1975–2024)
Source: Egypt Heart J. 2025 Sep 3;77:82. doi: 10.1186/s43044-025-00681-9 (PMC12408423; doi:10.1186/s43044-025-00681-9)
Supplement: Supplementary file 1 [file 43044_2025_681_MOESM1_ESM.docx]

**Supplementary figures:**

**Figure S1:** Annual scientific production of documents in antihypertensive medication adherence.


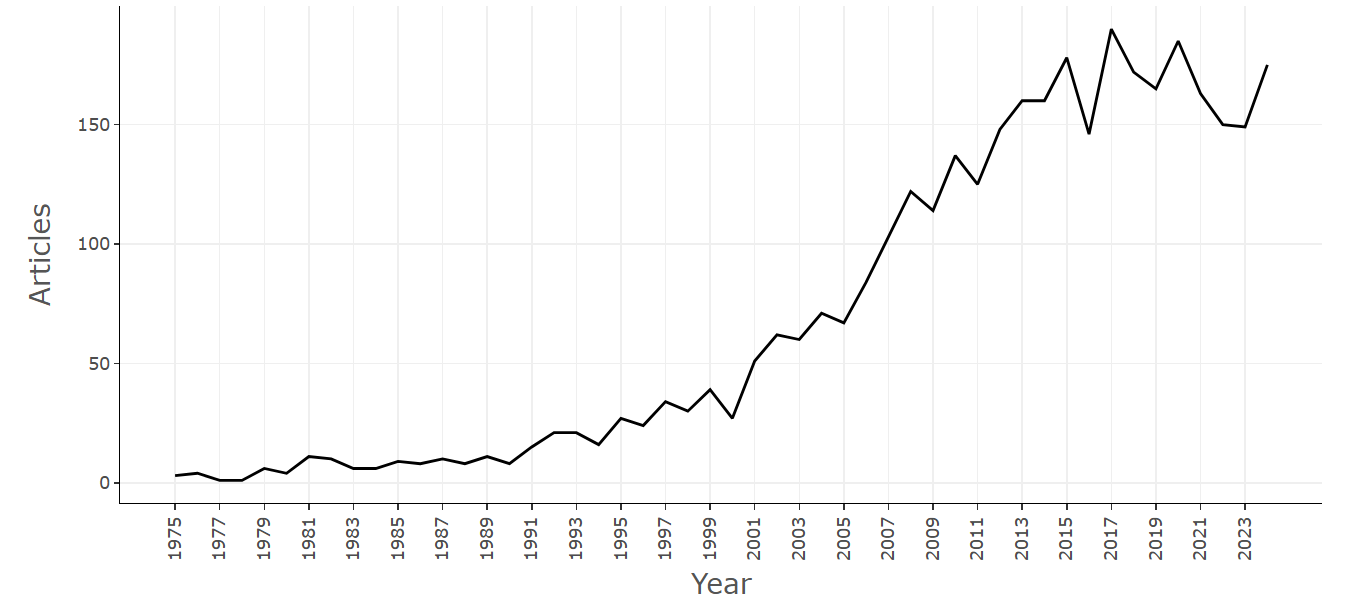
*Generated using the biblometrix package in the R programming language.

**Figure S2:** Annual citations per year in antihypertensive medication adherence research articles

**
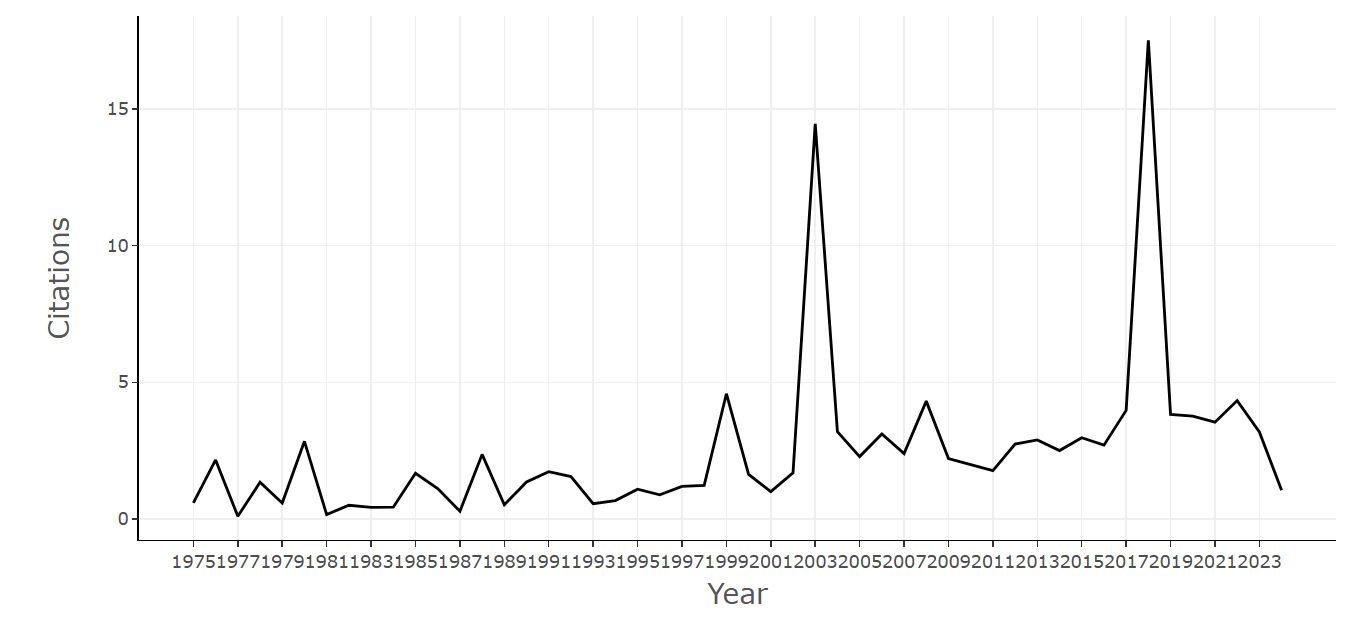
***Generated using the biblometrix package in the R programming language.

**Supplementary Table**

**Table S1.** The adjusted search terms as per searched electronic database (Scopus) [as of 21.12.2024]

| No | Scopus Search Query | Results |
| --- | --- | --- |
|  |  | (Till 21.12.2024) |
| #1 | (ti,ab,kw = (Adherence* OR Adherent OR Nonadherence OR Non-adherence OR Patient adherence OR Medication adherence OR Compliance ) | 504287 |
| #2 | (ti,ab,kw = Antihypertensive* OR Antihypertensive therapy OR Antihypertensive therapy OR Antihypertensive treatment OR Antihypertensive medication OR High blood pressure medication OR Hypertension treatment) | 20080 |
| #3 | **#1 AND #2** | **3599** |
| Filters applied | | |
| Publication period: Database inception to 21^st^ December 2024\| | | |
| Document type include: Article, Review, Book chapter, conference paper and short survey  Exclude: Retracted, Letter, Editorial, Note. | |  |

**Table S2: Top 10 most contributed countries in antihypertensive medication research**

| **Rank** | **Country name** | **Number of documents** |
| --- | --- | --- |
| 1 | United States | 1144 |
| 2 | United Kingdom | 269 |
| 3 | Germany | 246 |
| 4 | Italy | 233 |
| 5 | Spain | 184 |
| 6 | France | 162 |
| 7 | Canada | 159 |
| 8 | China | 155 |
| 9 | Australia | 147 |
| 10 | Netherlands | 131 |

**Table S3: Top 10** **affiliations of authors in antihypertensive medication adherence research**

| **Rank** | **Author’s affiliation** | **Number of documents** | **Total citations** | **C/A** | **h_index** |
| --- | --- | --- | --- | --- | --- |
| 1 | VA Medical Center | 67 | 5740 | 86 | 34 |
| 2 | Harvard Medical School | 51 | 2255 | 44 | 28 |
| 3 | The University of Alabama at Birmingham | 50 | 20137 | 403 | 23 |
| 4 | Università degli Studi di Milano-Bicocca | 48 | 1929 | 40 | 26 |
| 5 | Brigham and Women's Hospital | 48 | 2990 | 62 | 29 |
| 6 | Centre Hospitalier Universitaire Vaudois | 41 | 2795 | 68 | 23 |
| 7 | Université Paris Cité^a^ | 36 | 3205 | 89 | 19 |
| 8 | University of Pennsylvania | 40 | 4166 | 104 | 26 |
| 9 | Imperial College London | 40 | 3701 | 93 | 23 |
| 10 | AP-HP Assistance Publique - Hopitaux de Paris | 38 | 1923 | 51 | 18 |
